# Amyotrophic Lateral Sclerosis: Recent Considerations for Diagnosis, Pathogenesis and Therapy

**DOI:** 10.3390/brainsci15050498

**Published:** 2025-05-13

**Authors:** Andrew Eisen

**Affiliations:** Division of Neurology, University of British Columbia, Vancouver, BC V6T 1Z4, Canada; eisen@mail.ubc.ca

## 1. Introduction

Amyotrophic lateral sclerosis (ALS/MND) is considered a uniquely human complex neurodegenerative disorder, presenting with a variety of clinical phenotypes, which include frontotemporal dementia [1]. Cognitive impairment is common in ALS, and ALS and frontotemporal dementia (FTD) represent a disease spectrum unified by the presence of TDP-43-positive, ubiquitin-positive neuronal inclusions [2,3]. The heterogeneity of ALS (age of onset, site of initial clinical deficit, disease duration and rate of progression) is predicated by genetic, environmental, lifestyle and epigenetic determinants.

There is no naturally occurring animal ALS, and induced animal models whilst usefully mimicking anterior horn cell death—and to a lesser extent the demise of upper motor neurons—cannot truly recapitulate human ALS. This has resulted increasingly in the use of ALS patient-derived iPSCs, as shown in ***Publication 11***, which enables biological disease pathways, CRISPR gene editing, external risk factors and potential therapeutic agents to be studied. This methodology also allows us to investigate the early stages of ALS and tailor therapeutic responses. ALS is syndromic in nature and comprises a heterogeneous disease group, and successful therapy requires personalized targeting to tackle individual biological mechanisms of disease.

Heterogeneity in ALS is particularly evident in its overlap with frontotemporal dementia (FTD). Depending on the intensity of investigation, cognitive impairment is evident in >50% of ALS cases [4]. The expansion of FTD variations is ongoing, with the most recent labeled *right temporal variant frontotemporal dementia (rtvFTD)*. This variant is insidious in onset and may initially be mistaken for depression with subsequent apathy, language, and behavioral abnormalities. About 30% are associated with motor neuron symptomatology and pathology within the corticospinal tracts (***Publication 4***). 

## 2. Articles Published in the Special Issue

This Editorial is part of the Special Issue “*Amyotrophic Lateral Sclerosis: Recent Considerations for Diagnosis, Pathogenesis and Therapy*”. Twelve (12) manuscripts were accepted for, and all were subject to a rigorous review process. They are listed below.

1. Eisen, A.; Pioro, E.; Goutman, S.; Kiernan, M. Nanoplastics and Neurodegeneration in ALS. Brain Sci. 2024, 14(5), 471; https://doi.org/10.3390/brainsci14050471.
2. Correia, J.; Gromicho, M.; Pronto-Laborinho, A.; Oliveira Santos, M.; de Carvalho, M. Creatine Kinase and Respiratory Decline in Amyotrophic Lateral Sclerosis. Brain Sci. 2024, 14(7), 661; https://doi.org/10.3390/brainsci14070661.
3. Calma, A.; van den Bos, M.; Pavey, N.; Santos Silva, C.; Menon, P.; Vucic, S. Physiological Biomarkers of Upper Motor Neuron Dysfunction in ALS. Brain Sci. 2024, 14(8), 760; https://doi.org/10.3390/brainsci14080760.
4. Kleinerova, J.; McKenna, M.; Finnegan, M.; Tacheva, A.; Garcia-Gallardo, A.; Mohammed, R.; Tan, E.; Christidi, F.; Hardiman, O.; Hutchinson, S.; Bede, P. Clinical, Cortical, Subcortical, and White Matter Features of Right Temporal Variant FTD. Brain Sci. 2024, 14(8), 806; https://doi.org/10.3390/brainsci14080806.
5. Ozdinler, P. Sleep Apnea and Amyotrophic Lateral Sclerosis: Cause, Correlation, Any Relation?. Brain Sci. 2024, 14(10), 978; https://doi.org/10.3390/brainsci14100978.
6. Shandiz, E.; Fernandes, G.; Henkin, J.; McCombe, P.; Trajano, G.; Henderson, R. Assessing the Effect of Riluzole on Motor Unit Discharge Properties. Brain Sci. 2024, 14(11), 1053; https://doi.org/10.3390/brainsci14111053.
7. Ferullo, L.; Risi, B.; Caria, F.; Olivieri, E.; Poli, L.; Gazzina, S.; Leggio, U.; Bertella, E.; Giovanelli, G.; Labella, B.; Padovani, A.; Filosto, M. Gold Coast Criteria in ALS Diagnosis: A Real-World Experience. Brain Sci. 2024, 14(11), 1055; https://doi.org/10.3390/brainsci14111055.
8. Rocha, P.; Bento, N.; Svärd, H.; Lopes, D.; Hespanhol, S.; Folgado, D.; Carreiro, A.; de Carvalho, M.; Miranda, B. Voice Assessment in Patients with Amyotrophic Lateral Sclerosis: An Exploratory Study on Associations with Bulbar and Respiratory Function. Brain Sci. 2024, 14(11), 1082; https://doi.org/10.3390/brainsci14111082.
9. Pongrácová, E.; Buratti, E.; Romano, M. Prion-like Spreading of Disease in TDP-43 Proteinopathies. Brain Sci. 2024, 14(11), 1132; https://doi.org/10.3390/brainsci14111132.
10. Donaghy, R.; Pioro, E. Neurophysiologic Innovations in ALS: Enhancing Diagnosis, Monitoring, and Treatment Evaluation. Brain Sci. 2024, 14(12), 1251; https://doi.org/10.3390/brainsci14121251.
11. Dawoody Nejad, L.; Pioro, E. Modeling ALS with Patient-Derived iPSCs: Recent Advances and Future Potentials. Brain Sci. 2025, 15(2), 134; https://doi.org/10.3390/brainsci15020134.
12. Eisen, A.; Kiernan, M. The Neonatal Microbiome: Implications for Amyotrophic Lateral Sclerosis and Other Neurodegenerations. Brain Sci. 2025, 15(2), 195; https://doi.org/10.3390/brainsci15020195.

## 3. Early Disease and Preclinical Opportunities

ALS progresses in a multistep process, the seeds of which may originate at conception or in the peri-natal period [5]. Aside from genetic factors, during embryogenesis, motor neurons and their supporting glia are susceptible to many other potential insults, including neuroinflammation, excitotoxicity, mitochondrial dysfunction, excessive oxidative stress and external environmental risk factors. Epigenetic influences further determine individual sensitivity and susceptibility. Together, these factors are encompassed in the ALS exposome (***Publication 1***) [6]. It is recognized that these early insults can impact neurodevelopment but can also cause neurodegeneration in later life, as illustrated in ***Publication 12***. Although research is still in its infancy, there is a vital need to develop early and robust preclinical markers for ALS. Creatine kinase (CK) catalyzes the reversible conversion of creatine and adenosine triphosphate (ATP) to phosphocreatine and adenosine diphosphate (ADP) and is an essential component of ATP recycling and energy metabolism. Raised CK levels are associated with poorer respiratory outcomes (***Publication 2***). The preclinical window is potentially long with the opportunity to protect at-risk neurons and prevent already dysfunctional ones from demise [7].

## 4. Excitement About Excitotoxicity

A highly flexible neural network is essential to normal brain functioning. Throughout life, this is dependent on an optimal excitatory/inhibitory (E/I) balance controlled, respectively, by glutamatergic and GABAergic systems. Impaired E/I balance has been implicated in several neurodegenerations, including ALS [8]. Altered E/I balance is difficult to measure, but ***Publications 3 and 10*** explore this issue. Glutamatergic excitatory neurons far outnumber GABAergic inhibitory interneurons across all cortical circuits (with a ratio of approximately 80% to 20%). Despite their minority presence, inhibitory interneurons are a major determinant of network function and regulation and are key to their formation, plasticity and the rewiring of inter-neuronal synaptic contacts, which enables the fine-tuning of pyramidal neuron output activity. The defective development of GABAergic interneurons is likely the key to excitotoxicity in ALS and studies are needed to determine which class of interneuron is primarily responsible.

## 5. The Hallmark TDP-43

TDP-43 is a key area of ALS research, aspects of which were explored in ***Publication 9***. Although TDP-43 pathology is the hallmark of ALS, occurring at a frequency of 97%, mutations in the gene encoding TDP-43 occur in less than 1% of all ALS cases [9]. This implies that non-genetic factors converge to impact TDP-43 functioning, displacement and aggregation in ALS, particularly when related to nucleocytoplasmic shuttling. TDP-43 aggregation is a late-stage event, whereas partial mis-localization or complete nuclear depletion of TDP-43 occurs early in ALS. Upper motor neuron (Betz cell) cytoplasmic TDP-43 is soluble and has been postulated to transmit corticofugally with aggregation in spinal motor neurons. However, the biology of TDP-43 cell-to-cell spread is undetermined, with some evidence supporting prion-like spread (***Publication 9***).

## 6. The Essence of Good Sleep

Sleep is critical to brain functioning and disrupted sleep is linked to neurodegeneration [10]. Sleep disturbances may be identified years or even decades prior to overt disease in Alzheimer’s disease and Parkinson’s disease. Sleep abnormalities are well documented in ALS but only once the disease is clinically overt. The glymphatic system provides a pathway for the clearance of toxicants, including cytoplasmic TDP-43 and glutamate excess, and increased CSF levels of TDP-43 occur in ALS patients, implying that the system is not properly cleared (***Publication 5***) [11]. The prolonged impairment of meningeal lymphatics alters E/I balance with synaptic dysfunction. Flow is propelled by arterial pulsation, respiration, and posture, as well as the positioning and proportion of aquaporin-4 channels (AQP4). Non-REM slow-wave sleep is key to glymphatic drainage, which slows dramatically during wakefulness.

## 7. Essential Diagnostic Criteria

Diagnostic criteria are essential to the successful recruitment for ALS clinical trials and clinical care programs. Despite their shortcomings, the revised El Escorial criteria are still widely used. The Awaji–Shima criteria were developed to improve on the El Escorial criteria. Because of difficulties and inherent limitations to both sets of criteria, the World Federation of Neurology, the International Federation of Clinical Neurophysiology, the International Alliance of ALS/MND Associations, the ALS Association (United States), and the Motor Neuron Disease Association convened a consensus meeting (Gold Coast, Australia, 2019) to consider the development of simpler criteria that better reflect clinical practice [12]. The Gold Coast Criteria consistently have greater sensitivity than the El Escorial or Awaji criteria, primarily because patients with exclusively lower motor neuron signs in two or more body regions are included. Thus, these criteria are increasingly being used (***Publication 7***).

## 8. Future Considerations

Within the last few years, our understanding of ALS has significantly evolved, and it is now recognized as having a diverse spectrum. The cause of selective vulnerability in specific neurodegenerations is key to our understanding but remains largely enigmatic [13]. ALS has long been linked to the corticospinal system, with early dysfunction and the loss of Betz cells [14]. These corticofugal projection neurons are central to the execution of an array of skilled tasks and exhibit monosynaptic connections with bulbar and spinal motor units; however, the motor units that subserve external ocular and bladder wall muscles are largely spared in ALS. Aging remains the greatest risk factor in the multistep process, culminating in the clinical syndrome. Many of the pathophysiological pathways identified as important in ALS are also shared by the aging nervous system [15].

Akin to other neurodegenerations, ALS is multigenetic. Further, genomics have revealed considerable genetic overlap amongst major neurodegenerations. Very few identified genes are “causative” and the >50 identified in relation to ALS have very limited effects and best regarded as “at-risk genes”. Gene interactions of at-risk genes influence each other through a variety of mechanisms, and the expression of one gene can affect the expression of others. These interactions can lead to complex phenotypes. There are multiple dysfunctional biological pathways underlying ALS pathogenesis, which is in large part responsible for the lack of effective ALS therapy, and successful treatment will likely require combination therapy targeting these multiple pathways, especially in those cases lacking a causative genetic mutation [16,17]. The successful treatment of ALS remains elusive, and existing therapies have yielded minimal impact. Currently, eight drugs are approved by the US Food and Drugs Administration (FDA), namely Qalsody (tofersen, #N215887); RELYVRIO (AMX0035, #N216660); Radicava™ (edaravone, #N209176); Rilutek (riluzole, now generic, #N020599); Tiglutik™ (thickened riluzole, #N209080); Exservan (riluzole oral film, #N212640); Nuedexta^®^ (#N021879); and, recently, tofersen, which is specific to patients with SOD1 mutations. 
